# Endomembrane Reorganization Induced by Heavy Metals

**DOI:** 10.3390/plants9040482

**Published:** 2020-04-09

**Authors:** Monica De Caroli, Antonella Furini, Giovanni DalCorso, Makarena Rojas, Gian-Pietro Di Sansebastiano

**Affiliations:** 1 Department of Biological and Environmental Sciences and Technologies (DiSTeBA), University of Salento, 73100 Lecce, Italy; monica.decaroli@unisalento.it (M.D.C.); makarena.rojas@unisalento.it (M.R.); 2 Department of Biotechnology, University of Verona, 37134 Verona, Italy; antonella.furini@univr.it (A.F.); giovanni.dalcorso@univr.it (G.D.)

**Keywords:** heavy metals, endomembranes, cell wall, remodeling, cellular trafficking, plant stress

## Abstract

Plant cells maintain plasmatic concentrations of essential heavy metal ions, such as iron, zinc, and copper, within the optimal functional range. To do so, several molecular mechanisms have to be committed to maintain concentrations of non-essential heavy metals and metalloids, such as cadmium, mercury and arsenic below their toxicity threshold levels. Compartmentalization is central to heavy metals homeostasis and secretory compartments, finely interconnected by traffic mechanisms, are determinant. Endomembrane reorganization can have unexpected effects on heavy metals tolerance altering in a complex way membrane permeability, storage, and detoxification ability beyond gene’s expression regulation. The full understanding of endomembrane role is propaedeutic to the comprehension of translocation and hyper-accumulation mechanisms and their applicative employment. It is evident that further studies on dynamic localization of these and many more proteins may significantly contribute to the understanding of heavy metals tolerance mechanisms. The aim of this review is to provide an overview about the endomembrane alterations involved in heavy metals compartmentalization and tolerance in plants.

## 1. Introduction

Heavy Metals (Pb, Zn, Ni, Cd, Fe, Cr, Cu, etc.) and Metalloids (As, Se, Sb) are naturally present in the Earth’s crust, some of them are essential for life such as Fe, Cu, Zn, Co, Mn, Mo, and Ni and are required as micronutrients that act as cofactors in biochemical reactions or play other physiological role into the cell and become toxic when in excess. Other elements, such as Cd, Hg, and Pb do not have biological functions and are toxic even if present at low concentration [1,2]. Here, for simplicity, the term Heavy Metals (HMs) refers to both heavy metals and metalloids. The content of HMs in the environment has increased in the last decades due to anthropogenic actions [3,4]. Indeed, mining and industrial activities, agricultural practices, and urban traffic release HMs into the environment. Such transition elements cannot be further degraded or destroyed and the exposition to high level is toxic to all living organisms including humans [5]. In addition, crop yield can be affected by excess HM in soils and plants represent the main entry pathway of HMs into the food chain. In the past decades, the use of model systems has led to a significant progress on the identification and characterization of candidate genes involved in HM movement and destination inside the plant cell, and on their role in physiological processes. These achievements and the future work investigating the molecular mechanisms underneath plant resistance to HMs will allow to produce healthy food and biofortified food crops with increased concentration of essential HM [6], and also to tailor plants ideal for phytoremediation [7].

Some species are defined metal hyperaccumulators because they are naturally able to accumulate extremely high concentrations of HMs in their aerial tissues [8]. This peculiar trait occurs without toxicity symptoms and hence it implies hypertolerance [9,10]. The most extreme example is represented by *Sebertia acuminata,* a Ni hyperaccumulator species in which the Ni content in latex can reach 26% of the dry weight [11]. Two hyperaccumulator species *Arabidopsis halleri* and *Noccaea caerulescens* (formerly *Thlaspi caerulescens*) are suitable models for physiological and genetic analyses of the metal hyperaccumulation trait thanks to their high similarity and extensive synteny with *Arabidopsis thaliana* [12]. Both species hyperaccumulate Zn and Cd (although with certain differences among populations); and *N. caerulescens* ecotypes endemic on serpentine soils are also Ni hyperaccumulator [13]. Another hyperaccumulator species identified more recently is represented by the fern *Pteris vittata* [14]. This fern can hyperaccumulate As and it represents an interesting model system to investigate As metabolism in plants [15]. During the past decades, a variety of molecular approaches brought to the identification of candidate determinants controlling the hyperaccumulation trait. This process requires, indeed, increased metal uptake and xylem loading as well as enhanced metal accumulation and detoxification in shoots. A breakthrough emerged from comparative physiological and molecular analyses of hyperaccumulators and related metal sensitive plant varieties, highlighting that the hyperaccumulation trait does not rely on a new resistance mechanism acquired during evolution, but it rather depends on the different regulation and activity diversification of proteins which are involved in metal handling, accumulation, and detoxification (e.g., metal transporters, chelators, and proteins involved in stress responses) [15,16,17,18].

In recent years, increasing attention has also been paid to the effect on the environment of nanoparticles, atomic or molecular aggregates with nano-dimension (between 1 and 100 nm), an inevitable product of volcanic natural events or accidental industrial applications [19]. Engineered nanoparticles, for example silver nanoparticles (AgNPs), are used in agriculture as plant growth and fruit ripening stimulators [20] or as fungicides [21]. Plants can absorb metallic nanoparticles present, for example, in heavy metal laden effluents from industrial sources [22]. Plants can also accumulate metallic species in the shape of nanoparticles, as observed in hydroponic studies. In those conditions, Gardea-Torresdey [23,24] first demonstrated the presence of metal nanoparticles of gold and silver in leaves of alfalfa phytoaccumulator species. Equally, the presence of nanoparticles of silver with size close to 50 nm has been observed in *Brassica juncea* and *Medicago sativa* [25]. Likewise, the uptake of heavy metals in the form of nanoparticles has been evidenced by plants grown autogenously in polluted areas. In leaves of *Dittrichia viscosa* and *Cichorium intybus*, the presence of nanoparticles was observed using dynamic light scattering, X-ray fluorescence, and transmission electron microscopy [26].

Up to now, research has deeply investigated the physiological and molecular aspects of the HM effect and transport into the plant body or the plant cell, but only little information has been published upon the effects of HM on the organization of the cell endomembrane system. It appears clear that this extended compartment could play a role in the turnover of particular transporters, influencing their activity and therefore effect on plant tolerance towards HM stress. Therefore, we aim here to give an overview of the morphological alteration showed by endomembrane compartments upon stress due to excess metal ions.

## 2. Metal Ions Movement Across Membranes

Plants are able to transport metal ions according to their growth and development, dispatching them to growing organs and to the cell compartments where they are integrated into biological functions. For instance, a fine control of metal trafficking is required in energy-handling organelles, such as chloroplasts and mitochondria, in which metal ions are involved in electron transport, but could cause serious oxidative damage [27]. Toxic HMs are also embedded into metal transport through the plant tissues and body. A variety of fine mechanisms are enacted by plants to survive and to maintain a functional cell metabolism in the presence of toxic HMs. These can be aimed either to avoid or tolerate the stress. Avoidance includes the mechanisms protecting the plant cell from HMs entering the protoplast, while tolerance is accomplished by those mechanisms enabling the plant to neutralize toxic metals (or their toxic effects) or to remove them from sensitive compartments (by sequestration in the apoplast or in the vacuole). In this context, metal transporters assume a pivotal role, being transmembrane proteins able to accomplish metal transport across biological membranes. These proteins are involved in root uptake, root-to-shoot translocation, and maintenance of cellular metal homeostasis by distributing metal ions into/out of cellular organelles and compartments. There is a great dynamism in metal trafficking into the cells and the plant body, which is mainly due to: (i) the fact that sharing similar chemical proprieties, some essential and toxic HMs share also the same uptake pathways; (ii) related transporters may compete for cation transport, thanks to their broad substrate affinity; (iii) members of the same transporter family may show different tissue expression and/or subcellular localization [28]. Metal transporters have been divided into families, according to sequence homology. The main families of metal transporters which are involved in the uptake of metals into the cytosol (influx transporters), or in its compartmentalization into cellular compartments and organelles or extrusion from the cellular environment (efflux transporters) are ZIPs, natural resistance-associated macrophage protein (NRAMPs), cation diffusion facilitator (CDFs), and heavy metals ATPase (HMAs), but also members of YSL, CAX, and MATE transporter families are involved [29].

Members of the ZIP family (Zinc–Iron Permease, also known as ZRT, IRT-like proteins) are implicated in the transport of a variety of metals, including the essential Fe, Zn, Cu and Mn, and the toxic Cd [1,30]. Their name is due to the first three described members of this family, involved in transport of Zn in yeast and Fe in *A. thaliana*, ZRT1 and ZRT2, and IRT1 respectively. In *A. thaliana*, 18 sequences have been identified as putative members of the ZIP family, and their role is still under investigation. Some proteins seem to be involved in intracellular influx of ions and are located on the plasma membrane: AtZIP2, ZIP4, and ZIP9 in *A. thaliana* and ZNT (homologous to ZIP4) in *N. caerulescens* are likely to play a role in uptake of Zn, Mn, and Cd [30,31]. Interestingly, Fe transporters such as IRT1 and IRT2 are able to take up Cd in both *Oryza sativa* and *A. thaliana* [32]. Other ZIP transporters may be responsible for metal distribution into cellular compartments, as AtIRT2, which is located in endo-membranes vesicles contributing to Fe transport into these compartments [33,34] and OsZIP1, showing a dual location on the plasma membrane and on the endoplasmic reticulum upon normal growth conditions and limiting Zn, Cu, and Cd accumulation in rice tissues [35].

Metal transporters that belong to the natural resistance-associated macrophage protein (NRAMP) family have been shown to transport a variety of metals across membranes, and as the members of the previously mentioned ZIP family, generally display poor selectivity towards divalent metal cations [36]. Generally characterized by ten to twelve transmembrane domains with a predicted transport motif between the VIII and IX transmembrane helices, NRAMP transporters are described to have broad metal specificity with the same protein able to interact with few metals. The metal transport capacity of NRAMP proteins has been demonstrated by complementation of yeast mutants impaired in metal uptake [37]. Members of this family can reside in the plasma membrane, while others sit on the tonoplast, highlighting a possible function in adjusting cytosolic metal concentration or releasing metals from the vacuole [36]. In *A. thaliana* and *O. sativa*, there are 6 and 7 NRAMPs respectively. AtNRAMP1 is upregulated in the root under Mn deficiency and it is believed to be a high affinity Mn transporter with plasma membrane localization [29]. AtNRAMP3 and AtNRAMP4, as well as AtNRAMP1, can complement yeast deficient in either Mn or Fe uptake. They were localized in vivo on the tonoplast and are able to transport Fe, Mn, and Cd [38]. AtNRAMP4 was also identified as part of the vacuolar proteome in *A. thaliana* [39]. Less is known for AtNRAMP2, AtNRAMP5, or AtNRAMP6. The study of NRAMPs in rice shows that OsNRAMP3, but not OsNRAMP5, responds to environmental changes in Mn availability. OsNRAMP3 was localized on the plasma membrane and when Mn is in excess, it is internalized in vesicles to be degraded as part of a post-translational regulation in response to environmental nutrient availability [29].

The third large family of metal transporters is grouped under the name of cation diffusion facilitator (CDF), whose members are phylogenetically ubiquitous and have been identified in bacteria, yeast, plants, and mammals [40]. In plants, CDF proteins are also identified as metal tolerance proteins (MTPs) and are mainly involved in the efflux of cations from the cytoplasm through sequestration into internal compartments or through efflux from the cell [41]. Indeed, most MTPs are localized in the tonoplast, working as antiporters of Zn, Cd, and Ni [42] or in other subcellular compartments, such as AtMTP11, which resides within a punctate endomembrane compartment consistent with either *trans*-Golgi or pre-vacuolar organelles. Moreover, being highly expressed in the leaf hydathodes, AtMTP11 seems to be involved in vesicular trafficking and exocytosis of excess Mn at secretory tissues where Mn will be excreted rather than stored [29,41]. 

The last family of metal transporters that will be analyzed in this review is defined heavy metals ATPase (HMA) and groups P-Type ATPase proteins able to transport metal ions such as Ca, Cu, Zn, Cd, Co, Pb, and Mn across membranes upon ATP hydrolysis [43]. HMAs have been extensively studied in *A. thaliana* and *O. sativa*: their localization includes the plasma membrane, the tonoplast, and the cellular endomembrane system. HMAs are involved in delivering Zn and Cu in and out of the chloroplast and into plastid sub-compartments, in Zn and Cd xylem loading, in detoxification of Zn, Cd, Co, and Pb by pumping them into the vacuole [43]. Interestingly, two particular P-Type ATPases, AtECA1, and AtECA2 which are located in the endoplasmic reticulum and the Golgi apparatus, respectively, function in carrying Mn ions from the cytosol to the respective compartment [29].

Heavy metals cross membranes not only by transporters but also through the water channel called aquaporin (AQPs). AQPs belong to the family of Major Intrinsic Proteins (MIPs) [44] that transport other molecules of great physiological interest in addition to water [45] and are possibly engaged in structural roles [46,47,48]. In plants, MIPs form a large superfamily of proteins with more than thirty identified members in *A. thaliana* [49] and *Populus trichocarpa* [50]. 

Several studies report that AQPs are differentially expressed in response to HM stress, but patterns are not uniform among species. For example, HMs’ stress in *Solanum torvum* and *A. thaliana* induces a general down regulation of aquaporin transcripts [51,52,53], while in *B. juncea* induces an up-regulation of a PIP1 aquaporin able to improve HM resistance when over-expressed in transgenic plants [54]. Several studies report that aquaporins can undergo modifications, such as glycosylation [55] or ubiquitination [56], which accompany a stress-induced or development-induced re-localization. Once entered the cell environments, free cytosolic metal ions are sequestered into a particular compartment (e.g., the vacuole, an organelle of the endomembrane systems) or follow chelation reactions. The cell environment is rich in HM chelating compounds and molecules, represented by thiol-rich peptides, phytochelatins (PCs), organic acids, such as citric acid or malic acid, glutathione, histidine, and other amino acids, metallothioneins, and nicotianamine [57,58,59,60,61,62,63]. Alteration of endomembrane compartmentalization is then an important symptom of HM-related stress.

## 3. Metal Ions Effects on Membranous Compartments

### 3.1. Endoplasmatic Reticulum

A role of endoplasmic reticulum (ER) in HM tolerance has been well reported in yeast. In *Schizosaccharomyces pombe*, the Zhf (Zinc homeostasis factor) protein, located in the ER and in the nuclear envelope, detoxifies the cytosol from Zn ions by accumulating them in the ER. S. *pombe* zhf mutant cells have increased Zn and Cu sensitivity and, at the same time, increased Cd and Ni tolerance [28]. Gardarin and co-workers identified the ER as the principal target of Cd toxicity in Saccharomyces cerevisiae [64]. Additionally, mammalian cells show the induction of typical markers of ER stress after Cd exposition [65]. A number of CDFs located on ER membranes, e.g., mammalian ZnT2 and ZAT from *A. thaliana*, have been shown to mediate Zn tolerance as well as increased Zn accumulation [66]. In plants, the ER was reported to be important in Pb detoxification. In epidermal cells of *Allium cepa* Pb toxicity was reduced by its accumulation mainly in ER-derived vesicles in the cytoplasm [67]. Cd exposition did not induce the same ER-derived vesicles even if the ER was altered in comparison with control condition [67].

AtNIP1;1 is an aquaglyceroporin able to transport arsenite (As[III]) and antimony, recently localized in vivo on ER membrane [48]. Its knock-out mutant (nip1;1ko) is resistant to toxic concentrations of As[III] but tolerance to arsenite is not due to a reduced uptake through the PM since in two different and independent studies [48,68] nip1;1ko lines were shown to regularly uptake As(III). If nip1;1ko has no effect on As(III) uptake it must control compartmentalization of the metalloid. Indeed this mutant or transgenic lines expressing tagged variants of NIP1;1 showed altered profiles for other elements uptake such as a reduction for Cu(II) and an increase for Zn(II) [48], confirming a role in metals and metalloids homeostasis. NIP1;1 was shown to interact with the vacuolar SNARE SYP51 and it is possible that through this interaction it regulates the vacuolar detoxification capacity. A different explanation for ko mutant resistance to arsenite is that the most important targets of As(III) toxicity are in the ER. The morphological changes of this marker under the effect of HMs have not been characterized.

Sequestration of Mn in the ER is an important mechanism of tolerance in plants but also other membranes are involved [69]. Four ER-type calcium ATPases (ECAs) in *A. thaliana* (AtECA1–4) and three in rice (OsECA1–3), belonging to the Ca^2+^-ATPase subfamily, are localized in part to the ER [69] and in part to the Golgi compartments [69,70]. AtYSL4 and AtYSL6 are reported to be localized on internal membranes resembling the ER in *A. thaliana* but also to vacuole membranes. Other elements of Mn tolerance mechanisms are located on *trans*-Golgi network membranes and tonoplast [69]. It is evident that further studies on dynamic localization of these and many more proteins is necessary.

### 3.2. Golgi Apparatus 

HMs can reach the Golgi apparatus (GA) through the secretory traffic but also directly from the cytosol thanks to specific transporters [71]. The most studied transporters localized in the GA membranes are CDFs. These antiporters are mostly localized in the tonoplast but were also found in the GA. For example OsMTP11 from rice, HvMTP8.1, and HvMTP8.2 from barley, PtMTP11.1, and PtMTP11.2 from *P. trichocarpa* as well as BmMTP10 and BmMTP11 from *Beta vulgaris* sequester Mn into the GA [69]. AtMTP11 is involved in Mn tolerance and is localized to a punctate endomembrane compartment probably in the *trans*-Golgi, but not to the plasma membrane and vacuole where other MTPs are localized (ShMTP1, OsMTP8.1, and OsMTP8.2). Its overexpression increases tolerance to Mn toxicity [72]. A recent work on *O. sativa* used a tagged version of the transporter, MTP11:GFP to evidence co-localization with *trans*-Golgi markers and not with pre-vacuolar compartment (PVC) markers. Anyhow, knockout of MTP11 in wild-type rice did not affect tolerance and accumulation of Mn or other HMs but caused secondary effects on fertility [73]. Only as second mutation in mtp8.1 knock-out lines was able to induce Mn sensitivity. These proteins must be kept in the correct location. It is not yet clear if these mechanisms lead to exocytosis as shown by Peiter at al., [74] or to PVC and vacuole as shown by other authors working with higher concentrations of Mn [72]. 

Effect of HMs, or adaptation to them, can involve GA in different ways. Transcriptomic studies under Cd stress have shown in unicellular algae *Dunaliella*, the overexpression of genes involved in carbohydrate metabolic process and located in ER and GA [75]. Polysaccharides biosynthesis is important in algae to counteract salt and drought stress but must also be important to neutralize pollutants such as HMs by secreting extracellular polymers [76].

Morphological alteration induced by HMs at GA level can reveal a specific relation between this organelle and the HM transport or accumulation. Whether this relation is direct or indirect remains to be investigated case by case. Eleftheriou et al. [77] monitored in time the effect of Cr(VI) exposures in *A. thaliana* roots. Plastids, mitochondria, GA, and vacuoles suffered the most evident damage; ER, cytoplasm, nuclei, and cell walls showed intermediate damage. GA was shown to suffer an early effect on morphology starting with an interesting semi-circular conformation, enclosing cytoplasmic material and producing at the end of the process ring-like structures containing translucent material [77]. Once more it is not possible to define if these effects are directly induced by Cr or by Cr-induced production of ROS [77].

Cr(VI) stress (100 μM potassium dichromate (K_2_Cr_2_O_7_)) appeared to be specific: after 48 h Golgi bodies were severely disorganized, consisting of a single circular cisterna, residual, or swollen cisternae. Additionally, ER formed large aggregations of parallel or concentric, ribosome-bearing, non-swollen cisternae. Sometimes, amorphous electron dense material was trapped between ER cisternae [77]. With Golgi, also mitochondria and plastids morphology was drastically altered even if their double-membrane envelopes remained structurally intact [77].

When considering the alteration of GA, dictiosomes mobility associated to cytoskeleton has to be considered. In fact indirect effect on endomembranes may derive from cytoskeleton disruption, like in the case of Cd stress that perturb both endocytosis and exocytosis because it disrupts the “tracks” (actin filaments) for vesicle transport [78]. The effect of Cd on actin filaments was studied in *A. thaliana* root hairs. 5 μM Cd destroyed the arrangement of actin filaments, changing them from a longitudinal to a transverse array. With increasing Cd concentrations, longitudinal AFs completely disappeared. The alteration in actin cytoskeleton derives from alteration of Ca metabolism and transport because many cytoskeletal proteins are sensitive to changes in Ca. For example Ca can be replaced by Cd in the crystal structure of the Ca-dependent actin-severing protein gelsolin activating the association of gelsolin with actin cytoskeleton, with gelsolin’s severing properties [78]. A more recent study on Cd stress effects on *Picea wilsonii* pollen germination and tube growth [79] investigated the effects on ER, GA, and vacuoles morphology. The study apparently ignored the effects on cytoskeleton [78].

### 3.3. Multi-Vesicular Bodies 

Vacuolar traffic and autophagy are important in homeostasis and detoxification processes but are often interconnected to ER and GA plasticity and involve the formation of the so-called Multi Vesicular Bodies (MVBs) [80,81,82,83,84,85]. A microarray study in *Populus* stressed by Zn allowed the identification of an AQP of NIP type, AQUA1 [86], down-regulated in both leaf and root tissues, possibly with the effect to reduce transpiration, water/Zn uptake, and leaf growth, probably for protecting photosynthetic tissues and for enhancing poplar tolerance [87]. The localization of AQUA1-GFP was also studied in *A. thaliana* and *N. tabacum* [85] showing a diffused distribution but also showing association with markers such as Cherry-BP80 and RFP-ATG8f in large MVBs. Treatments with 100 μM and 200 μM Zn affected the localization pattern of AQUA1-GFP in transiently transformed protoplasts reducing the number of fluorescent structures increasing their size. The co-treatment with Zn seemed to influence the reaction to 10 μM of HgCl_2_. AQUA1 is a mercury sensitive aquaporin and the reduction of Hg dependent alterations in the presence of 200 μM of Zn and the significant increase in vesicles number in *A. thaliana* protoplasts under excess Zn, suggesting a possible protective role of this protein. Zn treatments caused AQUA1-related MVBs’ aggregation in response to Zn stress with the effect to remove aquaporins from the ER. This may reduce ER permeability to Zn or concentrate all the transport in the MVBs. A similar mechanism of re-localization was reported also for the human aquaporin-2 in response to hormone-signaling [88] and for PIPs in *A. thaliana* roots treated with NaCl and salicylic acid [89]. 

The formation of separated compartments is a general mechanism to contrast HMs stress in both plants and animals. When the induced compartments are related to Zn stress were named ’zincosomes’ [90,91]. In plants, these compartments evolve as pro-vacuoles and may be considered as separated vacuoles. In fact, Zn detoxification in *A. thaliana* appears mediated by specific vacuolar metal transporter such as AtMTP1 and AtHMA3 [92,93]. AtMTP1 is normally localized on tonoplast and mtp1 ko mutants does not form Zn-induced vesicles [94]. Despite the possibility to evidence morphological effects on MVBs, their formation is certainly strictly related to the vacuole.

### 3.4. Vacuole

Vacuoles are essential to cope with HMs stress but are involved in very different pathways. In general, vacuolar sequestration of HMs reduces concentration of HM ions in the cytosol alleviating HM toxicity affecting cytosolic biochemical reactions. The first functionally characterized CDF for Mn transport, ShMTP1, was isolated from a tropical legume with higher Mn tolerance and was localized to the tonoplast [69]. Overexpression of ShMTP1 confers Mn tolerance in yeast cells and *A. thaliana* via sequestration of Mn into the vacuoles [69,95]. AtMTP1 also functions as a Zn^2+^/H^+^ antiporter transporting cytosolic Zn^2+^ into vacuoles [96]. Similarly, others CDFs are localized in the tonoplast working as antiporters of Zn^2+^/H^+^, Cd^2+^/H^+^, and Ni^2+^/H^+^ [42]. 

Endomembranes react to the HM stress changing traffic dynamic and in the case of vacuoles they change shape and size. The effects of various HM ions (both essential and non-essential) were studied in different conditions. Zn caused tubular vacuoles to turn spherical within 15 min in the hyphae of a *Paxillus involutus* [97]. Four days exposure to a subtoxic concentration of Zn, approximately a threefold and sevenfold increase in vacuolar volume fraction occurred in *O. sativa* and *Triticum aestivum*, while a similar increase was absent in *Secale cereale*. The Zn-induced vacuolation was proposed to represent a compartmentalization mechanism to reduce toxicity [98]. Ultrastructural analysis by transmission electron microscopy revealed that a 3-days exposure of a unicellular alga *Chlamydomonas acidophila* to a concentration of Cd close to the EC50, induced to doubling the vacuolar volume with the appearance of vacuolar deposits of phosphate and Cd. Cu and Zn treatment did not induce the same effects, suggesting those are metal-specific responses [99]. Increased vacuolation after Cd (20 μM) treatment was observed in meristematic root cells of 3-day-old seedlings of *A. thaliana* [100]. The cellular morphometry of root tip cortical cells of *A. thaliana* transformed with GFP fused to a tonoplast protein was studied correlating Cd concentration and vacuolation [100].

The effect of Cd in different systems can be heterogeneous. Cd induced the formation of small vesicles in suspension-cultured cells of *Nicotiana tabacum* [101] or had no effect at all on endomembranes of radish leaves cells [102]. The heterogeneity may be due to genotypic or experimental differences and more variability occur due to the effect of different HMs (some reviewed in [81]). 

Endomembranes remodeling was studied in *A. thaliana* under Cr(VI) stress [77]. Several severe effects were observed: bulbous outgrowths of nuclei and plastids, intranucleoplasmic macrotubules, vesicular structures budding at the edges of ER cisternae, and the formation of lipid droplets in the cytoplasm or in close association with plastids.

In Cr(VI)-treated cells, most vacuoles retained their integrity but they contained several inclusions, the most usual of which were variously shaped aggregations of granular precipitates. Other inclusions were irregular, membrane-bound structures, containing very dense granular material. Other frequently reported effects of Cr stress refer to membrane injury, vacuole disruption, and cytoplasm dilution due to mixing with vacuolar contents [77].

## 4. Metal Ions and the Cell Wall

Cell wall is the first barrier against HM entry into plant cells. HM ions can bind to the functional groups of all cell wall components but most of divalent and trivalent metal cations are bound by polysaccharides abundant in carboxyl groups [62,103,104]. HMs accumulated and precipitated in the cell wall are efficiently inactivated [104,105]. 

The degree of methylesterification and acetylation of pectin decreases its affinity for HM. In *Silene paradoxa*, a copper-tolerant cultivar differentiated from a copper-sensitive cultivar is shown to have in the root a reduced pectin content associated with an increased methylesterification [106].

The growing tips of apical cells of *Funaria hygrometrica* protonemata showed cell wall thickenings induced by Pb^2+^. The thickenings were evidenced by specific immunolabelling. The most evident effect was the appearance high amount of low-esterified (JIM5 epitope) and unesterified (PAM1 epitope) homogalacturonans. Both compounds, absent in the cell wall of control protonemata, are able to bind and immobilize Pb²⁺. Furthermore, Pb^2+^ induced an increasing of internalization by endocytosis of low-methylesterified pectins, Pb bound, from the cell wall to the vacuoles of protonemata cells. Interestingly, the cell wall thickenings were separated from plasma membrane by callose layer, compound impermeable to HMs, likely in order to prevent the internalization of Pb pectins into the cell [62,107]. High concentration of HMs led the formation of thickenings in the cell wall of root cell of *Vicia faba*, with the peculiarity that the cells with HMs deposits only in thickened cell wall did not appear damaged, while those with HMs deposits within the cell was seriously damaged. These observations suggested that the cell wall is involved in protection events from HMs in plants [108,109]. 

There are several examples of metal accumulation in the cell wall. The common reed (*Phragmites australis)* is considered to be a plant with a high phytoremediation potential. When exposed to high Zn levels, the metal was accumulated mainly in the cell wall [105]. The moss *Scopelophila cataractae* uses pectins to bind Cu in the cell walls and it can normally grow in Cu excess condition without suffering symptoms of toxicity [110]. The hyperaccumulator *N. caerulescens* and the fern *Lycopodium japonicum* bind in the cell wall about 50% of HMs [111,112]. In the water plant *Elodea canadensis*, up to 70% of Cd can be bound to the cell wall [113].

Some species enhance lignin biosynthesis in response to HM exposure, suggesting that lignin in plant cell walls may play a role in sequestering HMs [114,115]. It was reported that Cu has a positive effect on the biosynthesis of lignin [116]. In *Panax ginseng* exposed to a high level of Cu, lignin biosynthesis was enhanced [117]. In *M. truncatula*, high levels of Al induced increased activity of peroxidases and lignin deposition correlated to root growth inhibition presumably due to cell wall stiffening as a consequence of increased lignin deposition [118]. 

Chemical changes due to HMs can be revealed by the interference with water absorption due in part to the inactivation of water channel proteins and in part to a decreased cell wall extensibility or elasticity derived by the cross-linking of the pectin carboxyl groups in the walls with heavy metals [119]. The chemical transformation of the cell wall is anyhow not so important in all those cases where resistant mutants missing some transporters or channels show to recover a normal growth. In the case of As(III) for example, root growth inhibition is not depending on cell wall since the mutant missing one AQP able to transport As (NIP1.1, independent from cell wall deposition or modification), is fully resistant and grows normally [48].

## 5. Conclusions

The specific mechanisms induced by HMs causing endomembrane remodeling are largely uncharacterized. The most studied phenomenon is the extension of vacuolar system and still the mechanism is unknown [81]. The lack of knowledge is surprising considering that many genes involved in vesicular traffic such as SNAREs [120] have been identified. Toxic HM ions interfere with the cellular and molecular machinery associated with membrane traffic but it is also true that membranes composition and organization contribute to cope with HM effects providing additional tools to a future improvement of phytoremediation approaches [121]. An increased knowledge on membrane markers behavior under the effect of HMs-related stress may provide new tools in the study of these relevant aspects of plant physiology. The study of endomembrane adaptation to HMs effect on aspect should receive attention in the context of HM stress.
